# Evaluating Vancomycin Monotherapy and Dual Therapy with Nifuroxazide for Medium–Severe Clostridioides Difficile Infection

**DOI:** 10.3390/antibiotics14040400

**Published:** 2025-04-14

**Authors:** Jasna Rahimić, Ervin Alibegović, Lana Lekić, Marijana Marković Boras, Amina Džidić-Krivić, Esma Karahmet Farhat, Emina Karahmet Sher

**Affiliations:** 1 Faculty of Medicine, European University Kallos, Tuzla, XVIII Hrvatske Brigade 8, 75000 Tuzla, Bosnia and Herzegovina; jasna.rahimic@eukallos.edu.ba; 2 International Society of Engineering Science and Technology UK, Nottingham NG7 1GN, UK; marijana.markovic.boras@fpmoz.sum.ba (M.M.B.);; 3 Department of Clinical Center, Tuzla University, 75000 Tuzla, Bosnia and Herzegovina; 4 Faculty of Health Studies, University of Sarajevo, Stjepana Tomića 1, 71000 Sarajevo, Bosnia and Herzegovina; 5 Department of Laboratory Diagnostic, University Clinical Hospital Mostar, 88000 Mostar, Bosnia and Herzegovina; 6 Department of Neurology, Clinical Hospital Zenica, 72000 Zenica, Bosnia and Herzegovina; 7 Faculty of Food Technology, Juraj Strossmayer University of Osijek, 31000 Osijek, Croatia; 8 School of Science and Technology, Nottingham Trent University, Nottingham NG11 8NS, UK

**Keywords:** *Clostridioides difficile* infection (CDI), modified therapeutic protocol, vancomycin, nifuroxazide and dual antibiotic therapy

## Abstract

Background: All currently used therapeutic protocols and drugs for *Clostridioides difficile* infection (CDI) treatment do not have a satisfying success and usually cost a lot. Objectives: To compare the efficacy of vancomycin monotherapy vs modified dual therapy with vancomycin + nifuroxazide as a therapeutic protocol for a medium–severe form of CDI. In addition, the effects of a modified therapeutic protocol with standard monotherapy on the number of stools and stool consistency in a medium–severe CDI will be compared. Materials and Methods: A prospective, randomized, controlled clinical trial that included 60 patients divided into two groups was conducted. One group of patients was treated with vancomycin monotherapy. The other group was treated with the modified therapeutic protocol (vancomycin + nifuroxazide). Results: The modified therapy with vancomycin + nifuroxazide demonstrated enhanced pharmacological efficacy in the management of CDI compared to the standard vancomycin monotherapy. Patients treated with dual therapy reported a significantly lower number of stools in first, second and third control; first control (4.47 ± 2.20 compared to 5.70 ± 1.91 in vancomycin group (*p* = 0.024)), second control (2.37 ± 0.85 compared to 3.13 ± 0.90 in vancomycin group (*p* = 0.001)), and third control (1.53 ± 0.51 compared to 1.80 ± 0.61 in vancomycin group (*p* = 0.035)). Also, the first and third controls noted significant improvements in stool consistency, measured as a decrease in the number of completely watery stools (*p* = 0.011 and *p* < 0.001, respectively). Conclusions: Nifuroxazide and vancomycin have demonstrated accelerated improvement in patient status and hold promise as a novel dual therapeutic regimen for managing patients diagnosed with a medium–severe form of CDI.

## 1. Introduction

*Clostridioides difficile* is a Gram-positive sporogenous anaerobic bacterium commonly found in the human and animal gut as part of the normal microbiota [1]. Due to disruptions in the intestinal microflora, particularly following prolonged broad-spectrum antibiotic use, *Clostridioides difficile* infection (CDI) arises [2]. This infection presents a serious, life-threatening health condition, especially among geriatric and pediatric populations, as well as individuals experiencing gut microbiota dysbiosis. It accounts for approximately 20–30% of all diarrheal cases induced by antibiotic use [3] contributing significantly to morbidity and mortality and burdening healthcare systems worldwide [4].

Toxins produced by *Clostridium difficile* promote the onset of a series of complex cellular reactions in the host, resulting in diarrhoea, the production of inflammatory molecules and cytokines, as well as the necrosis of affected tissues [5]. Recently, there has been an increase in the incidence of moderate and severe cases of CDI, primarily due to the non-critical and often-avoidable use of broad-spectrum antibiotics that induce CDIs, as well as the occurrence of hypervirulent strains of *Clostridium difficile*, marked as NAP1/BI/027 [6]. The main features of this strain are the hyperproduction of *Clostridium difficile* toxins, the release of an additional virulence factor named the binary toxin, and the resistance to fluoroquinolone antibiotics, such as moxifloxacin. Studies have indicated that it shows significant resistance to other antibiotics like clindamycin and levofloxacin while also remaining susceptible to metronidazole and vancomycin [7].

Additionally, CDI exhibits a notable relapse rate, affecting around 20–30% of patients within the first two months after initial infection. Recurrences of infections represent an exhausting segment of the patient’s life due to repeated or frequent hospitalizations, increased healthcare costs, and often culminating in depressive states for affected individuals [8,9].

The infection can be mild, moderate, or severe. Mild and medium–severe forms of infection occur more often than severe ones. An essential sign of CDI is diarrhoea that occurs 5–10 days after the administration of antimicrobial therapy, and at least three or more watery stools are noted for at least two consecutive days [3]. Such a phenomenon is called post-antimicrobial diarrhoea, which can have the characteristics of mild to severe diarrhoea. The above-mentioned symptoms occur much more often and at a higher intensity in geriatric patients due to their health condition, immunity, and age. In the severe form of the disease, initial diarrhoea leads to the onset of pseudomembranous colitis, followed by an increase in the patient’s body temperature up to 38–38.5 °C, hypoalbuminemia, an increase in the leukocyte count, abdominal pain, fatigue, gastrointestinal bleeding, and dehydration [1]. In the case of more complex symptoms, pseudomembranous colitis is diagnosed, and the clinical presentation is difficult to predict because it depends on the virulence of the causative agent, the immune system, and the patient’s clinical response [10].

Besides the previously mentioned symptoms, it includes the presence of watery stools for 10 to 15 days and the possibility of kidney function damage. The most severe form of the disease occurs when pseudomembranous colitis turns into toxic megacolon, sepsis, which leads to a final, fatal outcome [11]. Smits et al. [12], reported that fulminant infection with *Clostridioides difficile* has a mortality rate of up to 80% or requires patients to undergo a total abdominal colectomy. Following the confirmation of CDI by laboratory stool testing and the assessment of its severity, medical treatment can be applied [4]. One of the significant challenges associated with CDI treatment is the dual role of antibiotics, which serve as both a risk factor for CDI and a primary treatment modality. This significantly narrows the treatment options [1]. The first step of therapy is to stop antibiotic therapy if the infection is caused by antibiotic use, followed by administering some of the indicated antibiotics [13]. According to recent guidelines, metronidazole alone is no longer recommended for initial treatment. Currently, available antibiotics recommended by the Infectious Diseases Society of America (IDSA) (Arlington, VA, USA) and Society for Healthcare Epidemiology of America (SHEA) (Arlington, VA, USA) for the treatment of CDI are vancomycin and fidaxomicin (recommended for the first episode and first reoccurrence) [14].

Considering fidaxomicin’s high cost, vancomycin is commonly used in clinical practice [15]. In case of mild–moderate and severe infection, vancomycin 125 mg 4 times a day for a minimum of 10 days is recommended [14]. Suppose the patient does not respond to any of the applied antibiotics. In that case, a faecal microbiota transplant (FMT) is recommended, which involves transferring the faecal microbiota from a donor with healthy gut microbiota (in the form of a faecal suspension) into the patient’s intestine to establish the normalization of intestinal flora [16]. Even though this procedure shows promise, it also has its limitations. This is why the FDA has published safety alerts, as there is concern about the potential transmission of pathogenic and multi-drug-resistant organisms (US FDA, Silver Spring, MD, USA, 2020).

Nifuroxazide is commonly employed in the treatment of acute and chronic bacterial diarrhoea. It alters bacterial enzyme activity, inhibits protein synthesis, and displays anti-inflammatory properties [17]. Remarkably, over 99% of orally administered nifuroxazide remains in the intestines, limiting systemic absorption and minimizing the risk of systemic side effects. It acts primarily as an intestinal antiseptic since its antibacterial action is expressed locally, preventing oxidative stress and exhibiting bacteriostatic effects at lower doses and bactericidal effects at higher doses [18,19]. However, it has been proven over time that it works by reducing the production of bacterial toxins and that, in therapeutic doses (800 mg/24 h), nifuroxazide does not disturb the complex homeostasis of the human intestinal flora [19]. It is also considered to have an exceptional safety profile because nifuroxazide does not lead to the onset of bacterial resistance [20]. A review of the literature showed good tolerance of nifuroxazide therapy and, in general, an absence of complications during treatment without adverse effects on faecal microbiota [17,21].

All currently used therapeutic protocols and drugs for CDI treatment do not have a satisfying success and usually cost a lot. Therefore, this study aims to investigate the effectiveness of a modified therapeutic protocol for CDI, including a dual therapy with vancomycin (4 × 125 mg) + nifuroxazide (4 × 200 mg) for 10 days compared to vancomycin monotherapy in the treatment of a moderate form of CDI. Given the complete absence of literary data on the use of nifuroxazide for CDI treatment, even in moderate to severe cases, this research represents the first study conducted regarding the use of this drug in CDI. Investigating this combination could contribute valuable insights into alternative treatment options for CDI, particularly in light of the ongoing challenges posed by antibiotic resistance and the need for effective, cost-efficient therapies.

## 2. Results

The baseline characteristics of both groups were similar: there was no difference in the age and gender of groups (*p* = 0.550 and *p* = 0.602, respectively). The incidence of males was 18 (60%) in the vancomycin and 16 (53.3%) in the vancomycin + nifuroxazide group. The median age in the vancomycin group was 73.16 ± 9.12 years (74.06 ± 9.70 for males and 71.83 ± 14.58 for females) and 74.17 ± 7.72 years (75.38 ± 7.26 for males and 72.79 ± 7.99 for females) in the vancomycin + nifuroxazide group.

Various parameters were measured upon admission and at each subsequent control for both groups. The results for the vancomycin-treated group are presented in Table 1, while those for the vancomycin + nifuroxazide-treated group are presented in Table 2. There was a significant difference between the results on admission and those of each subsequent control, indicating recovery in both groups. A gradual decrease in stool number was observed in both groups (*p* < 0.001). A change in stool consistency was observed, showing a decrease in the incidence of completely watery stools and an increase in the incidence of typical normal stools in both groups (*p* < 0.001 for both). The incidence of stools with blood decreased in both groups starting from the first control (*p* < 0.001 for both groups). The presence of stomach pain and pain intensity were also decreased (*p* < 0.001 for both measured parameters in both groups). The incidence of very strong severe cramps decreased in both groups (*p* < 0.001). A decrease in the number of hospitalized patients and the number of positive laboratory test results was observed in each consecutive control for both groups (*p* < 0.001).

**Figure 1:**
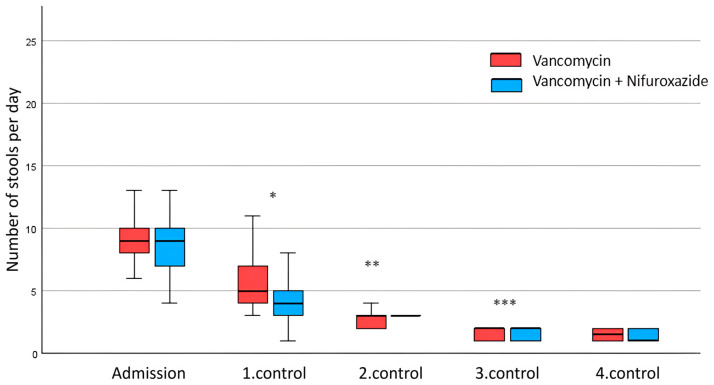
Comparative diagram of the number of stools in the group treated with vancomycin and the modified therapeutic protocol of vancomycin + nifuroxazide during all five control examinations. The graph shows the dispersion of results within each group. The black line in the middle represents the median. The coloured area presents results falling between the 25th and 75th percentiles. The horizontal end lines illustrate the range of results. Mann–Whitney U test for comparison between vancomycin and the modified therapeutic protocol of vancomycin + nifuroxazide (* *p* = 0.024; ** *p* = 0.001; *** *p* = 0.035).

However, there were noticeable variations in these changes between the two groups of patients. Therefore, the groups were compared to assess the impact of drug modality on the dynamics of recovery. The analysis of stool number data for each patient treated with vancomycin monotherapy revealed a gradual decline from 9.53 ± 3.19 stools upon admission to 1.50 ± 0.50 stools at the fourth control (*p* < 0.001). A similar phenomenon was observed in the vancomycin + nifuroxazide group, starting from 8.83 ± 4.06 stools on admission to 1.33 ± 0.48 stools in the fourth control (*p* < 0.001) (Table 1 and Table 2). There was no difference in stool number on admission (*p* = 0.461). However, as shown in Figure 1, the stool number was significantly reduced in the vancomycin + nifuroxazide group at the first control (4.47 ± 2.20 compared to 5.70 ± 1.91 in the vancomycin group; *p* = 0.024), the second control (2.37 ± 0.85 compared to 3.13 ± 0.90 in the vancomycin group; *p* = 0.001), and the third control (1.53 ± 0.51 compared to 1.80 ± 0.61 in the vancomycin group; *p* = 0.035).

**Figure 2:**
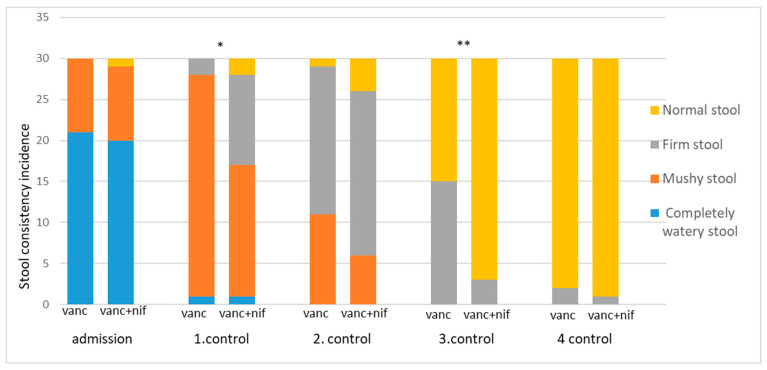
Stool consistency was categorized into four groups. The number of patients in each category was recorded on admission and on the day of 1. control, 2. control, 3. Control, and 4. control. (* *p* = 0.011; ** *p* < 0.001/χ^2^ test).

The stool consistency was assessed using a scale ranging from entirely watery stool (fluid or liquid consistency), mushy stool (soft but not completely liquid, may hold some shape), firm stool (well-formed and solid, but not excessively hard) to normal stool (moderately firm, typical consistency). The results are displayed in Figure 2. There was no difference in stool consistency between groups on admission (*p* = 0.599). In both groups, a gradual decline in the incidence of completely watery stool was observed, starting from admission (*p* < 0.001 for both groups). In the vancomycin group, upon admission, 21 (70%) stools were classified as entirely watery stools, whereas none were categorized as such by the fourth control. In the vancomycin + nifuroxazide group, 20 (66.66%) stools were classified as entirely watery stool upon admission, with none meeting this classification by the fourth control (Table 1 and Table 2). However, as shown in Figure 2, the vancomycin + nifuroxazide group exhibited an earlier improvement in stool consistency, with a significant increase in the number of firm stools observed at the first control (*p* = 0.011) and a substantial elevation in normal stool with typical consistency noted at the third control (*p* < 0.001) compared to the vancomycin group. Other measured parameters were also compared between groups on admission and at each control. No significant differences were observed. The following information can be downloaded from Supplementary Materials, presented in Table S1.

In the last stages of treatment (3. and 4. control check-up), a toxin test was performed to follow up on treatment success. The third control check-up showed a positive test in four participants, while in the last (4. control check-up), all participants had a negative toxin test.

## 3. Discussion

This study investigated the effectiveness of vancomycin monotherapy and vancomycin and nifuroxazide dual therapy in the treatment of medium–severe CDI in adults, revealing that dual therapy demonstrated superior efficacy in reducing the frequency of stools and improving stool consistency among these patients. The lack of significant differences in baseline characteristics suggests that patient demographics were adequately balanced between the treatment groups, reducing potential confounding factors in this analysis. CDI resolution (clinical cure) was defined as an absence of diarrhoea associated with CD following treatment cessation, indicated by either a maximum of three non-liquid stools (rated Bristol 5 or lower) per day or, in the event of diarrhoea, a negative CD test result [3]. Laboratory tests were not performed at the first and second control since conducting control CD testing during ongoing treatment with vancomycin is unreliable, as the sample may produce falsely negative results due to inadequate excretion of toxins [3]. Considering these rules, recovery in both groups of patients was evident, which is in line with previous research where vancomycin treatment was applied in the case of mild to moderate CDI [22].

A faster improvement in the clinical status of patients was evident in the vancomycin + nifuroxazide group. The results showed a tendency to decrease the number of stools per day in the first control (day 4) and primarily until the second control. Taking into account the results, which showed statistically significant differences in the comparison of successive controls of both groups and the most considerable difference analyzing the period between admission and the first control, it can be concluded that this modified therapeutic protocol significantly reduced the average number of daily stools in a short time for patients with a moderately severe form of CDI. A similar phenomenon was noted in research where the addition of nifuroxazide to standard metronidazole therapy also caused faster improvement in clinical status [23], proving the benefits of dual therapy. The results of the research conducted by Zar et al. in the period from October 1994 to June 2002 showed that at the applied dose of 250 mg, there was a complete improvement in the condition of patients suffering from CDI in 90% of cases, but there was an extremely long retention of symptoms, such as pains and cramps in the stomach and even increased body temperature with a very significant reduction in the number of daily stools [24].

Another significant observation was the improvement in stool consistency in patients treated with vancomycin + nifuroxazide compared to vancomycin monotherapy. In the vancomycin group, a significant improvement was observed only in the fourth control, as indicated by an increase in the number of stools with normal consistency. The slow effect of vancomycin on improving stool consistency was evident when it came to the therapy of a medium—severe form of infection. This is supported by the data visible in Figure 2. On the other hand, in the group of patients treated with the modified therapeutic protocol, the decrease in the number of watery and mushy stools was more rapid and could be seen even after the first check-up. The satisfactory effect of the therapy was consistently noted during each subsequent examination. This improvement reflects the faster restoration of gastrointestinal function and a reduction in CDI-associated diarrhoea [9]. The faster normalization of stool consistency in the dual therapy group underscored the benefits of combining nifuroxazide with vancomycin in managing CDI. Previous studies demonstrated that vancomycin was associated with a more significant reduction in stool frequency and a higher rate of clinical cures than metronidazole in treating CDI. Studies evaluating dual therapy of vancomycin and bezlotoxumab revealed similar cure rates but caused a decrease in reoccurrence of up to 10% [25,26].

A meta-analysis published in the Brazilian Journal of Infectious Diseases [27] that investigated the use of metronidazole and vancomycin in CDI therapy showed a slight recurrence rate, both with the use of vancomycin and metronidazole and both in the mild and moderate forms of infection. The mortality rate in this study was around 3% in a severe form of infection. The addition of other drugs, such as metronidazole, loperamide, bismuth derivatives, metoclopramide, and domperidone, to vancomycin in the treatment of both mild and medium–severe forms of the disease did not lead to faster elimination of pain and stomach cramps. The concomitant use of the mentioned drugs only led to the development of several side effects [27]. The effectiveness of vancomycin has also been confirmed by Erikstrup et al. [28] on experimental mice infected with *Clostridium difficile*. In this study, vancomycin administered at a dose of 4 × 125 mg over 10 days led to a significant halt in the loss of body weight in the experimental mice. The same authors concluded that the combined use of vancomycin and metronidazole did not give a better result in the treatment of CDI compared to the applied dose of vancomycin as a monotherapy.

Additionally, specific clinical parameters, including the incidence of stools with blood, the presence and intensity of stomach pain, and the incidence of very strong severe cramps, did not show significant differences between the two treatment groups in our study. These findings were consistent with previous research that has also reported similar incidences of these most common adverse events between different CDI treatment regimens [26]. While these parameters did not exhibit significant differences, their consistent trends across both treatment groups further validated the comparability of our study findings with existing literature. The results of this study have important clinical implications for the management of CDI. Current therapeutic protocols for CDI often lack efficacy and are associated with high costs. The findings suggest that the addition of nifuroxazide to the treatment regimen can enhance the pharmacological effectiveness of vancomycin, providing a more efficacious and cost-effective approach to treating medium–severe CDI. Moreover, the faster resolution of symptoms and normalization of stool consistency with the dual therapy regimen may lead to shorter hospital stays and improved patient outcomes. Despite the promising results, several limitations of the study should be acknowledged. Firstly, the sample size was relatively small, which may limit the generalizability of the findings. Future studies with larger sample sizes are warranted to validate the efficacy of the dual therapy regimen across diverse patient populations. Furthermore, the study did not investigate the mechanisms underlying the observed therapeutic benefits of the dual therapy regimen. Future research should focus on elucidating the pharmacological interactions between vancomycin and nifuroxazide to optimize treatment strategies for CDI.

## 4. Material and Methods

This prospective randomized study was conducted at the Department of Gastroenterology and Hepatology of the Clinic for Internal Diseases and Clinic for Infectious Diseases at the University Clinical Center Tuzla (Tuzla, Bosnia and Herzegovina) from June 2018 to June 2019. A total of 60 patients with CDI were included in the research and divided into two groups of 30 patients. A smaller sample size for this study was chosen due to the novel nature of the proposed treatment, which has not been previously tested in clinical settings. This careful approach ensures patient safety and enables us to monitor any unexpected adverse effects or outcomes closely. All patients were diagnosed as *Clostridioides difficile* positive by the proven presence of diarrhoea and a positive stool toxin test. The toxin test was performed in the last stages of treatment (third and fourth check-ups for follow-up). Diarrhoea is characterized by the presence of three or more watery or mushy stools per day (as per the Bristol Stool Form (ranging from the hardest to the softest) Scale 6 or 7) lasting for a minimum of two consecutive days. According to recommendations, infection was categorized as mild to moderate by the presence of diarrhoea and the absence of criteria present in severe or fulminant infections [3]. The inclusion criteria were age over 18 years, proven CDI using a toxin test, and classified as a medium–severe form of CDI, based on IDSA/SHEA 2021 guidelines [14]. The exclusion criteria were age under 18 years, severe or fulminant infection, heart failure classified according to NYHA (New York Heart Association), severe chronic obstructive pulmonary disease, grade III and IV renal insufficiency, and a stroke within a month before starting therapy.

Patients were divided into two groups. One group was treated with standard therapy with vancomycin 125 mg 4 times a day for 10 days, according to recent guidelines [14]. The other group was treated with a modified therapy, which was the use of vancomycin (4 × 125 mg) + nifuroxazide (4 × 200 mg) for 10 days. The dietary nutrition regimen was uniform for all patients. The monitored parameters were: number of stools per day, stool consistency (categorized as; completely watery stool (fluid or liquid consistency), mushy stool (soft but not completely liquid, may hold some shape), firm stool (well-formed and solid, but not excessively hard), normal stool (moderately firm, typical consistency), the presence of blood in the stool (Yes, No), number of hospital-observation days, the intensity of stomach cramps (4 categories: from very severe stomach cramps to absence of cramps), stomach pain (Yes, No), stool test for *Clostridioides difficile* (positive, negative), elevated body temperature (presence or absence) and intensity of stomach pain (4 categories: from very high to low pain intensity).

The distinction between the two parameters, stomach cramps and intensity of stomach pain, can be achieved by having both provided complementarily (Supplementary Materials). Pain intensity was described using the pain scale described by Haefeli [29]. Pain intensity on a scale from 1 to 10 allows for the precise quantification of pain. The scale allows the patient to express how painful their current sensation is, enabling an objective assessment based on subjective experience. For example, pain rated 1–3 would be “mild”, 4–6 “moderate”, 7–9 “severe”, and 10 represents the “worst possible pain”. Classifying pain as mild, moderate, severe, or no pain provides additional insights into how the patient perceives pain at specific time intervals during the course of treatment. These two parameters complement each other. Pain intensity provides a numerical value for the severity of pain, while the parameters of stomach cramps offer a more detailed insight into how the patient experiences pain, thus giving a fuller explanation of the pain the patient feels. In data analysis, both parameters could be used together to understand the patient’s pain experience and expression comprehensively, but we left them as separate parameters in the interpretation.

Patients were observed for 30 days, and all listed parameters were recorded on the day of admission to the hospital and during the first control (4th day from the start of therapy), the second control (10th day from the start of treatment), the third control (14th day from the beginning of treatment), and fourth control (30th day from the start of therapy). During the first four days of therapy, we could determine the initial response to treatment and identify any potential side effects. The 10-day period provided insight into the progress or stagnation of the treatment, meaning that by the tenth day, we could assess whether the disease was evolving or stabilizing under the influence of the treatment. It also allowed for identifying potential complications or improvements; based on this, the therapy could be modified if necessary. The control on the 14th day evaluated the treatment’s long-term effects, helping to determine whether the patient was achieving a complete or satisfactory clinical response to the treatment.

The patients had no insight into the questionnaires, which the responsible medical staff filled out and managed. The research was conducted after the approval of the Ethics Committee of the University Clinical Center Tuzla (No: 02-09/2-82/24) and following the Declaration of Helsinki. The patients included in the study signed informed consent forms after receiving detailed explanations about the study, including its duration, follow-up procedures, potential drug side effects, and confidentiality of research data.

## 5. Statistical Analysis

Standard methods of descriptive statistics were used (mean ± standard deviation). The Kolmogorov–Smirnov test was used to assess the normality of data. The Mann–Whitney U test and the Kruskal–Wallis test were used to compare numerical values. Categorical variables were compared by the χ^2^ test or Fischer’s exact test. The results were considered significant if *p* ≤ 0.05. The data were analyzed using the SPSS statistical program (Statistical Package for the Social Sciences), version 23.0 (IBM Corporation, Armonk, NY, USA).

## 6. Conclusions

The results of this study provide valuable insights into the treatment of *Clostridioides difficile* infection. CDI is a significant burden for health care systems and decreases the quality of life in affected patients. Current therapeutic regimes used for the treatment are usually expensive, without satisfying efficacy and safety, with an approximately 25% relapse rate in the first two months after the initial infection. By comparing the effectiveness of vancomycin monotherapy to a modified dual therapy regimen with vancomycin + nifuroxazide, significant improvements in patient outcomes were observed in the case of dual therapy. While certain parameters did not show significant differences between the treatment groups, the dual therapy regimen led to a substantial reduction in stool frequency, noted already at the first control, with patients experiencing a decrease to 4.47 ± 2.20 stools compared to 5.70 ± 1.91 stools in the vancomycin monotherapy group.

Additionally, the dual therapy group exhibited an earlier improvement in stool consistency, with a significant increase in the number of firm stools observed in the first control and a significant elevation in normal stool consistency noted by the third control. The observed improvements in stool frequency and consistency remain clinically meaningful and highlight the efficacy of the modified dual therapy regimen, representing an effective potential protocol for medium–severe CDI. These findings have important implications for clinical practice and highlight the potential of combination therapy approaches for improving the management of CDI. Further research is needed to confirm these findings and explore the underlying mechanisms of action. Since their mechanism of action is unknown so far, a checkerboard assay could be used to assess the interaction between these two antimicrobial agents and whether their combined effect is synergistic, additive, or indifferent. Hence, future studies should investigate this drug combination further in larger clinical trials with more patients. In addition, novel drugs or combinations of previously used drugs that are safe and efficient for treating CDI infection should be researched and engineered.

## Figures and Tables

**Table 1:** Comparison of measured parameters in the vancomycin group.

	Admission	1. Control	2. Control	3. Control	4. Control	*p*
Number of stools per day (mean ± SD)	9.53 ± 3.19	5.70 ± 1.91	2.37 ± 0.85	1.80 ± 0.61	1.41 ± 0.49	<0.001 *
Stool consistency (N% of Completely watery stool)	21 (70%)	1 (3%)	0	0	0	*p* < 0.001 **
Presence of blood in stool (Yes; %)	12 (40.0%)	4 (13.3%)	2 (6.7%)	1 (3.3%)	1 (3.3%)	*p* < 0.001 **
Number of hospitalized patients (N; %)	30 (100%)	30 (100%)	17 (56.7%)	10 (33.3%)	2 (6.7%)	*p* < 0.001 **
Stomach cramps (Very strong severe cramps N; %)	7 (23.3%)	3 (10%)	0	0	0	*p* < 0.001 **
Presence of stomach pain (Yes; %)	27 (90%)	17 (56.7%)	11 (36.7%)	6 (20%)	1 (3.3%)	*p* < 0.001 **
Intensity of stomach pain (Very high; N%)	7 (23.3%)	2 (6.7%)	0	0	0	*p* < 0.001 **
Presence of temperature/> 37 °C (Yes; %)	15 (50%)	9 (30%)	7 (23.3%)	3 (10%)	1 (3.3%)	*p* < 0.001 **
Positive laboratory test (N; %)	30 (100%)	Not performed	Not performed	6 (20%)	2 (6.7%)	*p* < 0.001 **

* Kruskal–Wallis; ** χ^2^.

**Table 2:** Comparison of measured parameters in vancomycin + nifuroxazide group.

	Admission	1. Control	2. Control	3. Control	4. Control	*p*
Number of stools per day (mean ± SD)	8.83 ± 4.06	4.46 ± 2.20	3.13 ± 0.90	1.53 ± 0.50	1.33 ± 0.47	<0.001 *
Stool consistency (N% of Completely watery stool)	20 (66.7%)	1 (3.3%)	0	0	0	*p* < 0.001 **
Presence of blood in stool (Yes; %)	11 (36.7%)	2 (6.7%)	1 (3.3%)	0	0	*p* < 0.001 **
Number of hospitalized patients (N; %)	30 (100%)	30 (100%)	13 (43.3%)	6 (20%)	1 (3.3%)	*p* < 0.001 **
Stomach cramps (Very strong severe cramps N; %)	6 (20%)	1 (3.3%)	0	0	0	*p* < 0.001 **
Presence of stomach pain (Yes; %)	26 (86.7%)	14 (46.7%)	8 (26.7%)	3 (10%)	0	*p* < 0.001 **
Intensity of stomach pain (Very high; N%)	7 (23.3%)	1 (3.3%)	0	0	0	*p* < 0.001 **
Presence of temperature/> 37 °C (Yes; %)	14 (46.7%)	6 (20%)	4 (13.3%)	0	0	*p* < 0.001 **
Positive laboratory test (N; %)	30 (100%)	Not performed	Not performed	4 (13.3%)	0	*p* < 0.001 **

* Kruskal–Wallis; ** χ^2^.

## Data Availability

Data are not publicaly available do to ethical restrictions.

## Supplementary Materials

The following supporting information can be downloaded at: https://www.mdpi.com/article/10.3390/antibiotics14040400/s1, Table S1: Comparison of measured parameters between vancomycin and vancomycin + nifuroxazide group.
