# Supplementary material for: Evaluating Vancomycin Monotherapy and Dual Therapy with Nifuroxazide for Medium–Severe Clostridioides Difficile Infection
Source: Antibiotics (Basel). 2025 Apr 14;14(4):400. doi: 10.3390/antibiotics14040400 (PMC12024110; doi:10.3390/antibiotics14040400)
Supplement: Supplementary file 1 [file antibiotics-14-00400-s001.zip › antibiotics-3514098-supplementary.pdf]

Supplementary materials

Table 1S. Comparison of measured parameters between vancomycin and vancomycin + nifuroxazide group.

	Vancomycin/ Vancomycin + Nifuroxazide				
	**Admission**	**1. control**	**2. control**	**3. control**	**4. control**
Number of stools per day	0.461	0.024	0.001	0.035	0.197
Stool consistency	0.599	0.011	0.185	<0.001	1.000
Presence of blood in stool	0.791	0.671	1.000	1.000	1.000
Number of hospitalized patients	1.000	1.000	0.302	0.243	0.500
Stomach cramps	0.931	0.379	0.389	0.058	0.492
Presence of stomach pain	0.688	0.438	0.405	0.472	1.000
Intensity of stomach pain	0.797	0.500	0.191	0.488	1.000
Presence of temperature	0.796	0.371	0.371	0.119	1.000
Positive laboratory test	-	Not performed	Not performed	0.488	0.492
